# Evaluation of *Salmonella* Serotype Prediction With Multiplex Nanopore Sequencing

**DOI:** 10.3389/fmicb.2021.637771

**Published:** 2021-03-10

**Authors:** Xingwen Wu, Hao Luo, Feng Xu, Chongtao Ge, Shaoting Li, Xiangyu Deng, Martin Wiedmann, Robert C. Baker, Abigail Stevenson, Guangtao Zhang, Silin Tang

**Affiliations:** ^1^Mars Global Food Safety Center, Beijing, China; ^2^Center for Food Safety, University of Georgia, Griffin, GA, United States; ^3^Department of Food Science, Cornell University, Ithaca, NY, United States

**Keywords:** whole genome sequencing, Oxford Nanopore Technologies, multiplex, *Salmonella*, subtyping, foodborne pathogens, serotyping, food industry

## Abstract

The use of whole genome sequencing (WGS) data generated by the long-read sequencing platform Oxford Nanopore Technologies (ONT) has been shown to provide reliable results for *Salmonella* serotype prediction in a previous study. To further meet the needs of industry for accurate, rapid, and cost-efficient *Salmonella* confirmation and serotype classification, we evaluated the serotype prediction accuracy of using WGS data from multiplex ONT sequencing with three, four, five, seven, or ten *Salmonella* isolates (each isolate represented one *Salmonella* serotype) pooled in one R9.4.1 flow cell. Each multiplexing strategy was repeated with five flow cells, and the loaded samples were sequenced simultaneously in a GridION sequencer for 48 h. *In silico* serotype prediction was performed using both SeqSero2 (for raw reads and genome assemblies) and SISTR (for genome assemblies) software suites. An average of 10.63 Gbp of clean sequencing data was obtained per flow cell. We found that the unevenness of data yield among each multiplexed isolate was a major barrier for shortening sequencing time. Using genome assemblies, both SeqSero2 and SISTR accurately predicted all the multiplexed isolates under each multiplexing strategy when depth of genome coverage ≥50× for each isolate. We identified that cross-sample barcode assignment was a major cause of prediction errors when raw sequencing data were used for prediction. This study also demonstrated that, (i) sequence data generated by ONT multiplex sequencing can be used to simultaneously predict serotype for three to ten *Salmonella* isolates, (ii) with three to ten *Salmonella* isolates multiplexed, genome coverage at ≥50× per isolate was obtained within an average of 6 h of ONT multiplex sequencing, and (iii) with five isolates multiplexed, the cost per isolate might be reduced to 23% of that incurred with single ONT sequencing. This study is a starting point for future validation of multiplex ONT WGS as a cost-efficient and rapid *Salmonella* confirmation and serotype classification tool for the food industry.

## Introduction

*Salmonella* is a genus of rod-shaped, Gram-negative bacteria which has imposed great risk on food production and public health (GMA, 2009; Scallan et al., 2011; Oh and Park, 2017; EFSA and ECDC, 2019a,b). As one of the most common foodborne pathogens, non-typhoid *Salmonella* contributes the second most cases of infections in the U.S. (Tack et al., 2020). Contamination often results in significant financial loss throughout the food supply chain, which makes effective surveillance and control of this pathogen necessary. Although there are only two species of *Salmonella*, over 2,600 serotypes have been reported to date (Issenhuth-Jeanjean et al., 2014). Thus, accurate and rapid identification of *Salmonella* serotypes is important for efficient source tracking during incident investigation and *Salmonella* surveillance.

The widely used White-Kauffmann-Le minor scheme defines *Salmonella* serotypes mainly by 46 somatic (O) and 114 flagella (H1/H2) antigens, and an antigenic formula with specific combination of O and H antigens is used to differentiate serotypes (Grimont and Weill, 2007). Traditional serotyping methods that use this scheme involve the use of specific antisera (Herikstad et al., 2002), with some serotypes further defined by biochemical characteristics. Reliable serotyping with traditional methods is time consuming, and requires careful maintenance of a large number of different antisera (Wattiau et al., 2011; Shi et al., 2015). Several molecular methods have been developed to overcome the drawbacks of traditional serotyping methods (Foley et al., 2007; Wattiau et al., 2011; Diep et al., 2019); for example, PCR (Herrera-León et al., 2007) and microarray-based subtyping methods (McQuiston et al., 2011) have been widely used. Due to increasing utilization of next generation sequencing (NGS), *in silico* subtyping through whole genome sequencing (WGS) data is gradually becoming mainstream. Several *in silico* methods have been developed to predict *Salmonella* serotypes from WGS sequencing data (Zhang et al., 2015; Ashton et al., 2016; Yoshida et al., 2016; Zhang et al., 2019), among which SISTR (Yoshida et al., 2016), SeqSero (Zhang et al., 2015), and its recent upgrade SeqSero2 (Zhang et al., 2019) have been substantively validated and shown to deliver accurate predictions (Diep et al., 2019; Banerji et al., 2020; Cooper et al., 2020). Such methods are now being accepted and used by regulators, industry, and academia for source attribution, outbreak investigation, surveillance, and research purposes (Didelot et al., 2016; EFSA Panel on Biological Hazards et al., 2019).

Illumina sequencing platforms^1^, which use short read sequencing approaches have been used commonly to provide WGS data for surveillance of foodborne pathogens, including *Salmonella* (Allard et al., 2016; Ashton et al., 2016; CDC, 2006, 2018). While Illumina sequencing has advantages of low error rate and high throughput, its short reads have limited capability for the closed assembly of genomes. Oxford Nanopore Technologies (ONT) offers comparable sequencing platforms with extra-long reads, real time sequencing, and rapid processing time^2^, but has higher rates of sequencing errors than Illumina (Fox et al., 2014; Rang et al., 2018). Raw ONT reads are long enough to be treated as “contigs” by SeqSero2, and this serotype prediction tool offers two options for ONT data: (i) raw ONT reads using SeqSero2 raw reads workflow (ii) raw ONT reads using SeqSero2 assembly workflow (Zhang et al., 2019). In SeqSero2 v1.1.2^3^, a single nanopore workflow is available for both raw ONT reads and their genome assemblies. This workflow algorithmically unifies the processing of both ONT raw reads and ONT assemblies by taking advantage of the long lengths of ONT reads, which are usually similar to those of contigs assembled from short reads (i.e., Illumina reads). With the serotype prediction tool SISTR, ONT raw reads have to first be assembled to correct base call errors, which have been reported to compromise SISTR prediction results (Zhang et al., 2019). Our previous evaluation of ONT sequencing based on sequencing a single *Salmonella* strain per flow cell and data analyzed by both the SeqSero2 raw reads workflow and SISTR revealed that a total of 2 h of ONT sequencing data were sufficient for successful *Salmonella* serotyping (Xu et al., 2020). This represents a considerable time saving compared to short-read-sequencing-based approaches. The ONT platforms have the capability for simultaneously sequencing multiple strains by applying DNA indexing (multiplex sequencing) (Karamitros and Magiorkinis, 2018). Combined with the continuously growing sequencing data yield of ONT sequencers, multiplex sequencing allows large quantities of complexed samples to be sequenced in one run (Piper et al., 2019; Kennedy et al., 2020). Several studies have successfully performed species identification by Multi Locus Sequence Typing (MLST) analysis from multiplexed sequencing data using the multiplex sequencing system provided by ONT (Imai et al., 2020; Liou et al., 2020). However, mis-assignment or cross-contamination of barcodes was also observed (Xu et al., 2018).

This study aimed to evaluate the efficiency, accuracy, and economic value of serotype prediction from ONT sequencing data generated from multiplexing up to ten *Salmonella* isolates. A combination of common, rare, and difficult-to-differentiate isolates were selected to (i) investigate whether correct antigenic formulae are assigned to isolates, (ii) determine whether mis-assignment or cross-contamination occurs during the process, and (iii) find the best combination of isolate number for multiplexing and the total sequencing time for the most efficient and accurate serotype prediction.

## Materials and Methods

### Bacterial Strains

Ten *Salmonella* isolates representing 10 different serotypes were assessed in the current study (Table 1). Seven isolates represented some of the most common serotypes, which included *Salmonella* serotype Typhimurium (including Typhimurium O5−), 4,[5],12:i:-, Paratyphi B (dt +), Enteritidis, Mbandaka, and Senftenberg. These were selected from i) the top 20 serotypes reported by the U.S. national *Salmonella* surveillance system (CDC, 2006, 2018) and ii) the top 20 serotypes causing human infections worldwide as reported to the WHO (Galanis et al., 2006). One isolate representing a rare serotype (serotype Havana) found in the food industry (information obtained by personal communication) was also included. Six isolates represented six serotypes that may be difficult to identify with molecular-level serotyping methods such as MLST and Pulsed-Field Gel Electrophoresis (PFGE). These six serotypes included (i) serotype Typhimurium, its O5^–^ variant, and serotype 4,[5],12:i:-, which are difficult to differentiate by MLST and phylogenetic analysis due to their close relatedness (Ranieri et al., 2013); (ii) serotype Paratyphi B (dt +), which has been incorrectly predicted by other molecular subtyping methods such as PFGE (Bailey et al., 2002; Soyer et al., 2010; Zou et al., 2012), and (iii) one strain of *S. enterica* subspecies *salamae*, and one strain of *S. bongori*; these two strains represent subspecies and species distinct from the most common *Salmonella* subspecies (i.e., *S. enterica* subspecies *enterica*). Detailed isolate information, including all sequence data associated with a given isolate, can be found at www.foodmicrobetracker.com under the isolate ID (e.g., FSL S5-0393).

**TABLE 1:** *Salmonella* strains used for evaluating the performance of multiplex ONT sequencing for serotype prediction from whole genome sequencing data.

**No.**	**Category**	**Strain ID**	**Recorded serotype^a^**	**Antigenic profile^b^**	**Source**	**Barcode no.^c^**
1	Most common	FSL S5-0483	Enteritidis	1,9,12:g,m:-	Human	07
2		FSL R8-4405	Mbandaka	6,7,14:z10:e,n,z15	Human, clinical	02
3		FSL S5-0658	Senftenberg/Dessau	1,3,19:g,[s],t:-	Human	03

4	Common and difficult to differentiate serovars (genetically similar)	FSL R9-3346	Typhimurium	1,4,[5],12:i:1,2	Human, clinical	01
5		FSL R8-3714	Typhimurium (O5^–^)	1,4,[5],12:i:1,2	Human, clinical	05
6		FSL S5-0580	4,[5],12:i:-	4,[5],12:i:-	Bovine	04

7	Common, and difficult to predict serovar correctly	FSL S5-0447	Paratyphi B (dt +)	1,4,[5],12:b:1,2	Human	08

8	Species and subspecies that are distinct from common *Salmonella* subspecies (i.e., *S. enterica* subspecies *enterica*	FSL R9-0514	*S. enterica* subsp. *Salamae*	55:z39:-	−	10
9		FSL R9-0518	*S. bongori*	66:z41:-	ATCC 43975	11
10	Rare	FSL S5-0549	Havana	1,13,23:f,g,[s]:-	Bovine	09

*^a^Food Safety Lab, Cornell University, provided the information of recorded serotype for each isolate, which was determined by conventional serotyping method with Kauffman-White scheme*

*^b^We listed the antigenic profiles of the strains in use based on the serotype information from “Antigenic Formulae of the Salmonella Serovars 2007 9th edition” (Grimont and Weill, 2007)*

*^c^The barcode No. 01–11 correspond to the barcode RB01–RB11 in the ONT Rapid Barcoding Sequencing kit (SQK-RBK004).*

### Genomic DNA Extraction

All *Salmonella* isolates were cultured on Trypticase Soy Agar at 37°C for 20∼22 h. Genomic DNA was extracted from single colonies using the QIAamp DNA mini kit (Qiagen, Hilden, Germany). The DNA quality and quantity were assessed with the NanoDrop 2000 (Thermo Fisher Scientific, Delaware, United States) for absorbance value (A) and the Qubit 3.0 fluorimeter (Life Technologies, Paisley, United Kingdom) for double strand DNA quantity, based on the guidance for qualification requirements for successful sequencing provided by ONT. The genomic DNA samples that met the following criteria were used for library construction: (i) A 260/280 between 1.8 and 1.9; (ii) A 260/230 between 2.0 and 2.2. The total input DNA was about 400 ng for each flow cell (FC).

### Oxford Nanopore Library Preparation and Sequencing

**FIGURE 1:**
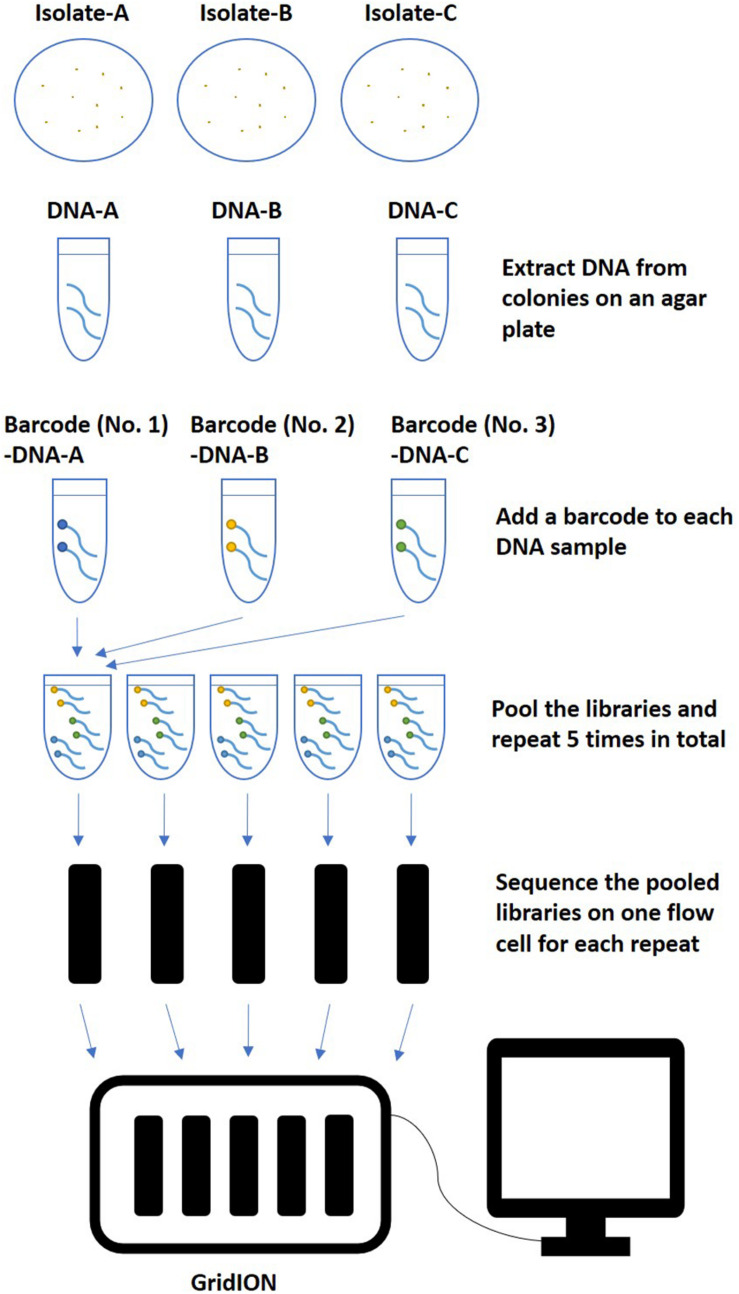
An example of the workflow of multiplex ONT sequencing library preparation and sequencing. After extracted from colonies on an agar plate, *Salmonella* genome DNA of three isolates were multiplexed and sequenced in one flow cell with five repeats. The same workflow was also used for multiplexing four, five, seven, or ten isolates in one flow cell.

The rapid Barcoding Sequencing kit (SQK-RBK004) was used according to the manufacturer’s instructions. Libraries were multiplexed and sequenced with qualified FLO-MIN106 flow cells (R9.4.1, active pore number ≥800) for 48 h on GridION (Oxford Nanopore Technologies, Oxford, United Kingdom) following the workflow described in Figure 1. Basecalling was performed in real time using Guppy with a basecalling model modified for 6 mA dam/5 mC dcm and CpG, which was integrated in the MinKNOW software v3.5.40 installed on GridION. Ten barcodes were assigned to ten isolates (Table 1). Five multiplexing strategies were tested, including multiplexing three (barcode No. 01 ∼ No. 03), four (barcode No. 01 ∼ No. 04), five (barcode No. 01 ∼ No. 05), seven (barcode No. 01 ∼ No. 05 and No. 07 ∼ No. 08), or ten (barcode No. 01 ∼ No. 05 and No. 07 ∼ No. 11) isolates on one flow cell. Repeats of each multiplexing strategy were tested on five flow cells, which were sequenced simultaneously on one GridION.

### Genomics Analysis and Data Distribution Analysis

Basecalled reads were demultiplexed using qcat v1.1.0^4^, the argument “–min-score” was set to a default value of 60 for general analysis, and various values were manually defined for the purposes of error analysis. Demultiplexed reads were classified based on the barcode qcat identified, reads of each barcode were applied to a modified genomic analysis workflow derived from our previous study (Xu et al., 2020). Porechop v0.2.3^5^ was used to trim adaptor sequences, and the quality of trimmed data was assessed using NanoPlot v1.18.1 (De Coster et al., 2018). A total of approximately 500 Mbp of high-quality long reads (minimum length > 1,000 bp) were selected through Filtlong v0.2.0^6^. Filtered raw reads were subsequently applied to an initial serotype prediction by SeqSero2 v1.0.2^7^ under a preset of arguments specifically designed for ONT raw data. Wtdbg2 v2.4 (Ruan and Li, 2020)^8^ was then used for initial genome assembly followed by one round of correction through Racon v1.3.3 (Vaser et al., 2017)^9^.

To assess the influence of barcode and isolate (as one barcode was always assigned to one isolate in this study, we combined these two factors into one as the barcode/isolate factor) on the distribution of data yield among multiplexed isolates in each flow cell, a one-way analysis of variance (ANOVA) was carried out to compare if the proportion of data yield of each barcoded isolate was significantly different from the other barcoded isolates multiplexed in the same flow cell. As the data yield per isolate was diverse within one flow cell, we calculated the coefficient of variation for the proportion of data yield per multiplexed isolate in each flow cell to compare the degree of diversity of data yield per multiplexed isolate between different multiplexing strategies. A one-way ANOVA was carried out to compare if this coefficient of variation for a given multiplexing strategy (e.g., three isolates) was significantly different from the other multiplexing strategies (e.g., four, five, seven, and ten isolates). Statistical significance for the ANOVA was assigned at α = 0.05.

### Serotype Prediction Analysis

**FIGURE 2:**
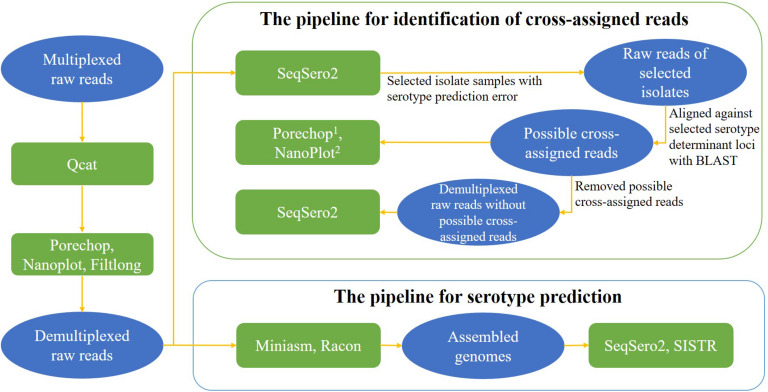
Data analysis pipelines for serotype prediction and identification of cross-assigned reads. Contents in ovals present the type of input data for each analysis, contents in rectangles present the tools for each analysis. ^1^Porechop was used to search for middle adaptors from the possible cross-assigned reads; ^2^NanoPlot was used to assess the sequencing quality of the possible cross-assigned reads.

The nanopore workflow available in SeqSero2 v1.1.2 (see text footnote 3) was used for genome assemblies from ONT reads for serotype prediction analysis. To avoid any possible impact of barcode cross-assignment on serotype prediction, the nanopore workflow of SeqSero2 v1.1.2 was not used on ONT raw reads for the purpose of serotype prediction. Instead, it was used to detect cross-assigned reads. ONT raw reads were also first assembled to correct basecall errors that were reported to compromise SISTR prediction (Zhang et al., 2019) and then used for further serotype prediction using SISTR_cmd (The *Salmonella in silico* Typing Resource Command-line Tool) v1.1.0 (Yoshida et al., 2016) under a default setting of arguments for assembled data. A brief data analysis pipeline for serotype prediction is shown in Figure 2.

#### Assessment of the Influence of Sequencing Time and Depth of Genome Coverage in Serotype Prediction

To assess the influence of sequencing depth and sequencing time on the accuracy of serotype prediction, serotype prediction analyses were carried out for each flow cell using different sizes of sequencing data collected as follows. The raw sequencing reads were arranged sequentially by their time of generation. For each flow cell, we first identified the isolate that accounted for the least amount of sequencing data, then we collected reads up to the time point when this isolate obtained the desired depth of genome coverage [average *Salmonella* genome size: 4.8 Mbp (McClelland et al., 2001; Parkhill et al., 2001)]. We defined this depth of genome coverage as Depth_min_ for this flow cell. For example, in FC01 (flow cell 01), sequencing data yield for isolates with BC01 (barcode 01), BC02, and BC03 after 48 h of ONT sequencing accounted for 29.36%, 34.95%, and 27.85% of the total sequencing data, respectively. If the desired Depth_min_ of FC01 for serotype prediction analysis was 15×, we collected sequencing data until the isolate with BC03 reached 15×depth of genome coverage (15 × 4.8 Mbp = 72 Mbp) since BC03 had the least share of sequencing data (27.85%) in this case.

#### Identification of Possible Cross-Assigned Reads and Influence of Cross-Assigned Reads on the Accuracy of Serotype Prediction

To further investigate if there were cross-assigned reads among multiplexed isolates on each flow cell, ONT raw reads was used as input of SeqSero2 to detect prediction errors caused by single ONT reads. Raw sequencing reads (at 99×depth of genome coverage) of the isolate that was predicted as a different serotype as recorded were aligned against selected serotype determinant loci using BLAST^10^. These antigen determinant loci were selected from the SeqSero2 antigen outputs that did not match the recorded antigen profile of this isolate. For example, the antigen profile prediction result using raw reads of isolate FSL R9-3346 from sample FC19-BC03 (recorded serotype: Senftenberg) showed its H2 antigen as “e,n,z15,” while serotype Senftenberg did not have an H2 antigen. We therefore included all four alleles of the H2 antigen “e,n,z15” from SeqSero2 database to align against the raw reads of FC19-BC03 for identifying possible cross-assigned reads. We defined a read as a possible cross-assigned read if it showed BLAST identity ≥90% and coverage = 100% to a selected antigen determinant allele.

Porechop v0.2.3 (see text footnote 5) was used to further assess whether there was a barcode in the middle of these reads (we choose a -middle_threshold of 60). Sequencing quality of these putative cross-assigned reads was assessed using NanoPlot v1.18.1 (De Coster et al., 2018). To assess the influence of the possible cross-assigned reads on the accuracy of serotype prediction, serotype prediction analyses with SeqSero2 were performed again after removal of these reads for the aforementioned two isolates. A brief data analysis pipeline for identification of cross-assigned reads is shown in Figure 2.

#### Assessment of the Influence of Sequencing Quality and Barcode Quality on the Accuracy of Serotype Prediction

To further assess the influence of (i) sequencing quality and (ii) barcode quality on the accuracy of serotype prediction for the two isolates FSL R9-3346 and FSL S5-0658 from the two samples noted in 2.4.2, serotype prediction analyses with SeqSero2 were performed with the raw reads selected with the following settings as input: (i) sequencing quality score ≥12, 13, or 14 (selected by Filtlong v0.2.0), qcat barcode score ≥60 (default setting), and Depth_min_ = 99×, as well as (ii) sequencing quality ≥7 (default setting), qcat barcode score ≥70, 80, or 90, and Depth_min_ = 99×.

## Results and Discussion

### Overview of Nanopore Sequencing Data and Assembly of *Salmonella* Genomes

The quality of raw sequencing data from the multiplex ONT sequencing experiments was analyzed by NanoPlot (version 1.18.1) (Table 2). An average of 10,629 Mbp of clean sequencing data per flow cell were obtained in 48 h from 25 experiments (across different numbers of multiplexed isolates). Data outputs of flow cells ranged from 5,650 to 17,998 Mbp, with the exception of FC17 which generated only 3,201 Mbp data; the mean read length and read length N50 varied much less (Table 2). Sequencing quality was shown to be highly consistent among 25 experiments, with mean quality scores for a given flow cell ranging from 10.8 to 12.3. The average quality score was 11.7.

**TABLE 2:** Summary of quality statistics of multiplexed raw sequencing data.

**Flow cell ID**	**Number of isolates multiplexed**	**Total clean data yield (Mbp)**	**Mean read length (bp)**	**Mean quality score**	**Number of reads**	**Read length N50**
01	3	13,787	10,368	11.7	1,329,726	18,407
02	3	12,312	8,025	11.5	1,534,118	14,802
03	3	13,694	7,583	11.2	1,805,906	15,665
04	3	11,085	8,207	11.3	1,350,613	15,284
05	3	8,135	8,385	11.3	970,123	15,235
06	4	14,463	8,305	11.7	1,741,442	14,596
07	4	6,332	8,017	11.7	789,817	14,266
08	4	17,100	8,283	11.8	2,064,501	14,731
09	4	15,234	8,226	11.2	1,851,964	15,137
10	4	17,998	7,658	11.8	2,350,186	13,968
11	5	13,715	7,832	10.8	1,751,213	14,332
12	5	7,181	8,018	11.5	895,600	14,680
13	5	8,761	7,600	11.3	1,152,794	14,648
14	5	10,530	7,583	11.1	1,388,584	14,689
15	5	6,833	7,434	11.6	919,206	13,320
16	7	13,513	7,167	12.2	1,885,531	13,479
17	7	3,201	7,831	12.1	408,795	14,618
18	7	8,450	7,542	12.1	1,120,392	14,249
19	7	12,113	7,706	12	1,571,937	14,447
20	7	5,984	8,212	12.3	728,703	14,714
21	10	5,650	9,429	12.1	599,172	17,944
22	10	9,666	9,851	12	981,235	18,013
23	10	7,794	9,370	12.3	831,812	17,038
24	10	10,592	9,770	12.1	1,084,229	18,057
25	10	11,603	10,309	12.2	1,125,520	18,549

Average		10,629.0	8,348.4	11.7	1,289,324.8	15,394.7

### Overview of Demultiplexed Sequencing Data

**FIGURE 3:**
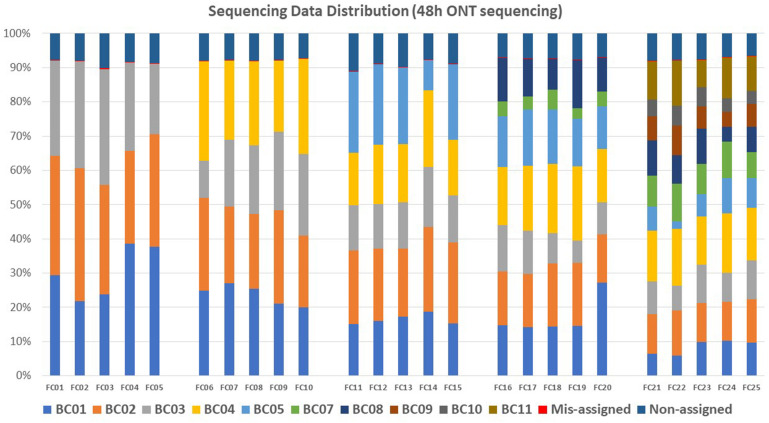
Sequencing data distribution within each flow cell (FC) for each barcode (BC). Each color represents one barcode. Within each flow cell, sequencing reads that were not assigned to any barcode were defined as non-assigned reads, reads assigned to a barcode that was not included in the flow cell were defined as mis-assigned reads.

An average of 8.02% (*N* = 25) reads per flow cell failed to be assigned to any barcode after demultiplexing analysis of the sequencing data generated with 48 h of ONT sequencing; these reads were defined as Non-assigned reads. An average of 0.03% reads per flow cell were assigned to barcodes that were not included in the flow cell; these reads were defined as mis-assigned reads (Figure 3 and Supplementary Table 1). Demultiplexed raw sequencing data of each multiplexed isolate in each flow cell from 48 h ONT sequencing (No. = 145) were submitted to NCBI–SRA (Accession number: PRJNA694442).

**FIGURE 4:**
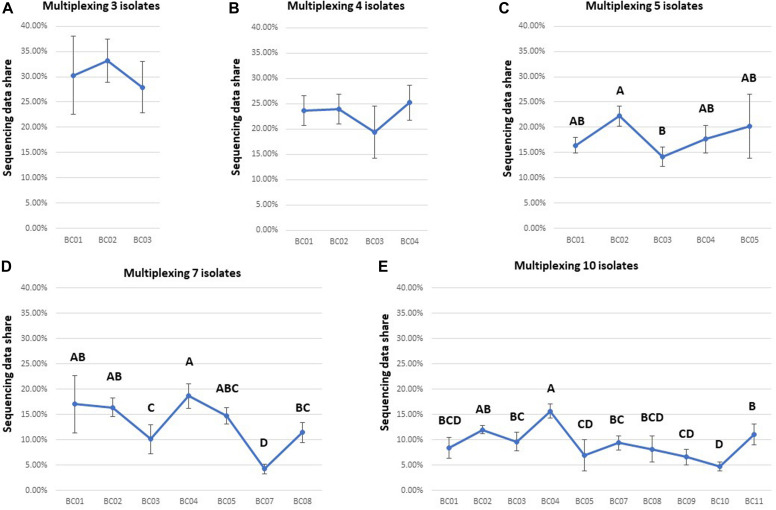
Comparison of the data yield of each isolate multiplexed in one flow cell for each multiplexing strategy (sequencing time: 48 h). Based on the statistical analysis results of Tukey HSD test, barcodes with different letters were significantly different (*P*-value < 0.05) from each other in data yield. Ideally, data share of each isolate was expected to be 31, 23, 18, 13, and 9% in **(A–E)**.

The unevenness of data yield among multiplexed isolates was a major barrier for shortening the total sequencing time for each flow cell. There was no significant difference among the data yields for different barcoded samples after 48 h of ONT sequencing when multiplexing three or four isolates (Figures 4A,B and Supplementary Table 1). However, ANOVA indicated a significant difference (*P* < 0.05) in data yields among isolates when multiplexing five, seven, or ten isolates (Figures 4C–E and Supplementary Table 1). For example, *post hoc* comparisons using the Tukey HSD test indicated that BC03 showed a significantly lower data yield (*P* < 0.05) than some other isolates when multiplexing five or seven isolates (Figures 4C,D), and BC07 showed a significantly lower data yield (*P* < 0.05) than some other isolates when multiplexing seven or ten isolates (Figures 4D,E).

**FIGURE 5:**
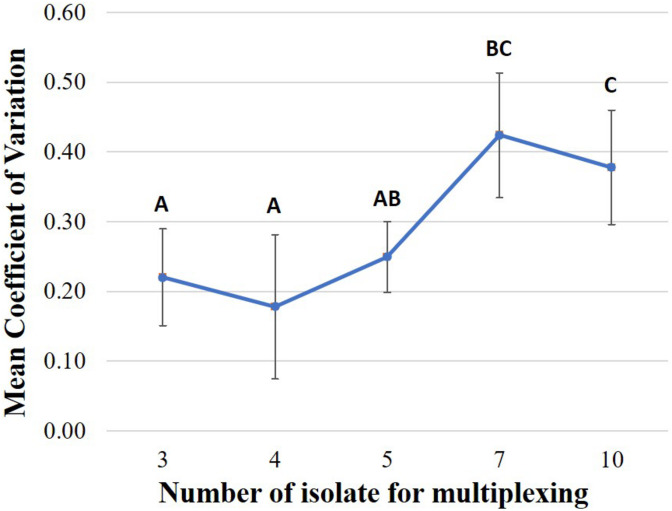
Comparison of the coefficient of variation of the proportion of data yield per multiplexed isolate in one flow cell under each multiplexing strategy including multiplexing three, four, five, seven, or ten isolates (Sequencing time: 48 h). Based on the statistical analysis results of Tukey HSD test, multiplexing strategies with different letters were significantly different (*P*-value < 0.05) from each other in coefficient of variation.

We also found that that the level of unevenness of data yield of each multiplexed isolate is different among multiplexing strategies (Figure 5). Comparison of the coefficient of variation of the proportion of data yield per multiplexed isolate among multiplexing strategies showed that, the level of isolate-data-yield variation of multiplexing seven or ten isolates is significantly greater (*P* < 0.05) as compared to multiplexing three, four, or five isolates. For example, on FC01 (three isolates multiplexed), the isolate (BC02) with the highest data yield accounted for 34.95% of total data (at 1,004×depth of genome coverage), which was 1.25 times the data yield of the isolate (BC03) with the smallest data sharing proportion (BC03 with 27.85% data yield at 800 × depth of genome coverage). On the other hand, for FC22 (ten isolates multiplexed), the isolate with the highest data yield (BC04) accounted for 16.72% of the total data (at 337 × depth of genome coverage); thus was 7.92 times the data yield of the isolate with the smallest data sharing proportion (BC05), which accounted for only 2.11% of the total data (at 42 × depth of genome coverage), after 48 h ONT sequencing.

### Influence of Sequencing Time and Depth on Accuracy of *Salmonella* Serotype Prediction Using ONT Assembled Genomes

For flow cells multiplexing three, four, or five isolates, five levels of Depth_min_ (15×, 30×, 50×, 75×, 99×) were used to perform serotype prediction analysis. Using genomes assembled from the raw reads with (i) sequencing quality ≥7 and (ii) qcat barcode score ≥60, both SeqSero2 and SISTR generated the same results at the serotype level as the recorded serotype of each isolate for all tested Depth_min_ levels when multiplexing up to five isolates. The ONT sequencing time for each flow cell at different Depth_min_ levels are shown below (Table 3).

**TABLE 3:** ONT sequencing time for different levels of Depth_min__/._

**Depth_min_ level**	**Sequencing time (hour)**					**Average Sequencing Time (hour)**
	**Multiplexing three isolates**					
	***FC01***	***FC02***	***FC03***	***FC04***	***FC05***	
99×	3.84	5.80	5.56	5.56	6.77	5.50
75×	2.93	4.41	4.25	4.25	5.09	4.19
50×	2.05	3.02	2.94	2.96	3.47	2.89
30×	1.31	1.93	1.90	1.90	2.19	1.85
15×	0.75	1.06	1.04	1.04	1.20	1.02

	**Multiplexing four isolates**					
	***FC06***	***FC07***	***FC08***	***FC09***	***FC10***	

99×	10.36	11.38	5.37	6.71	5.25	7.81
75×	7.81	8.43	4.10	5.10	3.99	5.89
50×	5.29	5.56	2.83	3.47	2.72	3.97
30×	3.27	3.34	1.82	2.14	1.72	2.46
15×	1.71	1.71	1.01	1.12	0.92	1.29

	**Multiplexing five isolates**					
	***FC11***	***FC12***	***FC13***	***FC14***	***FC15***	

99×	8.81	13.61	12.25	11.70	15.62	12.40
75×	6.66	9.84	9.01	8.71	11.35	9.11
50×	4.52	6.35	5.92	5.81	7.46	6.01
30×	2.79	3.69	3.62	3.58	4.50	3.64
15×	1.49	1.79	1.87	1.90	2.40	1.89

	**Multiplexing seven isolates**					
	***FC16***	***FC17***	***FC18***	***FC19***	***FC20***	

99×	33.35	−	>48	>48	>48	−
75×	21.75	−	24.09	41.82	>48	−
50×	13.62	−	12.99	20.37	31.18	19.54
30×	7.94	−	6.91	10.82	9.05	8.68
15×	3.96	−	3.35	5.16	4.00	4.12

	**Multiplexing ten isolates**					
	***FC21***	***FC22***	***FC23***	***FC24***	***FC25***	

99×	>48 h	>48 h	>48 h	>48 h	>48 h	−
75×	>48 h	>48 h	27.52	>48 h	31.01	−
50×	26.58	>48 h	13.20	20.81	17.06	−
30×	10.08	21.85	6.51	11.66	9.26	11.87
15×	4.13	9.54	3.16	5.60	4.41	5.37

For flow cells multiplexing seven or ten isolates, none of the flow cells achieved Depth_min_ = 99 × within 48 h of ONT sequencing. Hence Depth_min_ levels 15×, 30×, 50×, 75×, and/or the maximum Depth_min_ of each flow cell were used to perform serotype prediction analysis. One flow cell multiplexing seven isolates (FC17) generated only 3.20 Gbp trimmed data after 48 h of sequencing, which was 70% lower than the average total sequencing data; this flow cell was not included in the serotype prediction analyses. When multiplexing seven isolates, earliest accurate serotype predictions for tested isolates were obtained using sequencing data at Depth_min_ = 15×. When multiplexing ten isolates, SISTR identified isolate FSL S5-0483 (recorded serotype and antigen profile: Enteritidis, 1,9,12:g,m:-) as Gallinarum | Pullorum (predicted antigen profile: 1,9,12:-:-) in one (FC24) of the five replicates/flow cells, while SeqSero2 reported the correct serotype (with a depth of genome coverage of 41 × for this isolate in FC24). Earliest accurate serotype predictions for tested isolates were obtained using sequencing data at Depth_min_ = 15 × with SeqSero2 alone, and at Depth_min_ = 30 × with both SeqSero2 and SISTR.

While molecular serotyping does not typically identify serotype variants caused by ancillary O antigens, Typhimurium var. O5- (FSL R8-3714) was consistently (across flow cells) correctly identified by SeqSero2 [as SeqSero2 targets a mutation that causes the O5- phenotype (Hauser et al., 2011)]; SISTR, on the other hand, identified FSL R8-3714 as Typhimurium, without specifying the variant O5-.

In summary, for all multiplexing strategies tested in the current study, (i) with SeqSero2 alone, the earliest accurate serotype predictions were obtained using genomes assembled from sequence data at Depth_min_ 15 × as input, generated from 1.02 to 5.47 h of ONT sequencing for multiplexing three to ten isolates; (ii) with SISTR alone, the earliest accurate serotype predictions were obtained using genomes assembled from sequence data Depth_min_ at 30 × as input, generated from 1.85 to 11.87 h for multiplexing three to ten isolates (Table 3); (iii) both SISTR and SeqSero2 correctly predicted *Salmonella* serotypes from genomes assembled using multiplexed ONT raw data with 50 × genome coverage (please note that this is an actual coverage of a given isolate, not a Depth_min_ indicating the lowest genome coverage that each one of the multiplexed isolates can reach on one flow cell).

Higher quality of the assembled genome was shown to be associated with higher sequencing depth for both the WGS and ONT platforms in our previous study (Xu et al., 2020), while here we demonstrated a genome coverage of 50× is generally sufficient for assembling genomes to support accurate serotyping. An average sequencing duration of approximately 6 h for multiplexing five isolates was sufficient to reach this genome coverage. The majority of previous evaluations of WGS-based *Salmonella* serotype prediction used Illumina data (Zhang et al., 2015, 2019; Yachison et al., 2017; Uelze et al., 2020), since Illumina platforms can generally yield high quality sequencing data. Currently, a minimum of 30 × genome coverage (≥10 kb reads) of ONT sequencing data is recommended for bacteria assembly^11^.

Multiplexing several isolates inevitably caused variations in size of data allocation for each isolate, unlike sequencing a single isolate on a single flow cell. It is thus important to manage multiplex sequencing to allow each isolate to reach at least the genome coverage of 50 × for reliable serotype prediction, hence sequencing duration for multiplex runs will necessarily be longer than the sequencing duration required for a single isolate. Our results from pooling 3–10 isolates demonstrated that the unevenness of data yield among multiplexed isolates increased significantly and sequencing times required for accurate serotyping exceeded 19 h when multiplexing more than five isolates. Consequently, multiplexing more than five isolates may potentially undermine the benefit of the relatively short time required for ONT sequencing. Based on these observations, we conclude that multiplexing five isolates represents the optimum for obtaining the sequencing depth required for reliable serotype prediction within a reasonable time frame.

### Identification of Possible Cross-Assigned Reads and Their Influence on the Accuracy of Serotype Prediction

One and two possible cross-assigned reads were identified for samples FC10-BC01 (isolate FSL R9-3346; serotype: Typhimurium) and FC19-BC03 (isolate FSL S5-0658; serotype: Senftenberg), respectively (Supplementary Table 2). Please note that the analysis method we used could only identify possible cross-assigned reads leading to serotype prediction errors. Therefore, it was highly possible that the cross-assigned reads we identified were just a small fraction of all types of existing cross-assigned reads. The mean read length and sequencing quality of these reads were 10.96 Kbp and 12.93 Kbp, respectively. We did not detect any middle adaptors among these reads. These possible cross-assigned reads each had a barcode score ≥95.8 at one end of the read (Supplementary Table 2), and < 90 at the other end, as determined by Porechop (data not shown), consistent with the fact that the ONT library preparation kit added barcode adaptors to only one end of each read. Serotype predictions with raw reads by SeqSero2 after removal of these possible cross-assigned reads, were consistent with recorded serotypes of these samples, suggesting that these reads were the cause of serotype prediction errors for these two isolates. In addition, we did not identify any possible cross-assigned reads with our screening criteria from the corresponding isolates on the other flow cells multiplexing the same number of isolates but showing correct serotype predictions. As the barcode score and quality score of these detected cross-assigned reads were quite high and as no chimeric reads were detected (no evidence of middle adaptor), we speculated that one of the major causes of barcode cross-assignment was contamination from free adapters after pooling the libraries. These reads possibly captured wrong barcodes after multiplexing during library preparation. We did not perform cleaning-up to remove short sequences (< 100 bp) after pooling, hence these free barcodes might have had opportunities to link to the DNA sequences from multiple isolates. Tyler et al. (2018) also found erroneously barcoded reads that standard filtering practices could not remove as they were of high quality, and suggested running only a single sample at a time on a flow cell to avoid contaminating reads. It has been reported that clean-up by a bead-based or gel purification step could remove free adapters^12^, therefore a clean-up step after pooling the indexed libraries might be added in future studies. This may help alleviate index misassignment.

This study showed that cross-assigned reads might cause serotyping errors only when raw reads were directly used for serotype prediction. However, such errors may be avoided by assembling genomes prior to prediction. Barcode misassignment (including cross-assignment also known as index hopping) between multiplexed libraries is a recognized cause of misidentification (Kircher et al., 2012) for NGS. The Illumina sequencing technology has been reported to typically generate 0.1–2% barcode misassignment (see text footnote 12). Similarly, ONT sequencing has been reported to generate 0.02–0.3% barcode switching or misassignment to an unused barcode when using the 1D ligation sequencing kit (SQK-LSK108) and the native barcoding expansion kit (EXP-NBD103) (Tyler et al., 2018; Wick et al., 2018; Xu et al., 2018). In the current study, using the Rapid Barcoding Sequencing kit (SQK-RBK004), the index misassignment level was around 0.04% of the total sequencing data size (data not shown). Barcode misassignment levels for multiplex sequencing have shown to be highly dependent on the library preparation kit used, as well as quality and handling of the library (see text footnote 12). As the mean read length of the 25 ONT flow cells tested in the current study was above 8 Kbp (Table 2) and the length of an antigen determent loci in *Salmonella* is usually between 0.1 and 5 Kbp (data not shown), a small number of ONT cross-assigned reads can possibly alter the serotype prediction result. High molecular weight DNA extraction methods are available for purification of DNA in the 50 Kbp to 1 Mbp + size range. Reads with greater mean length may lead to higher quality of genome assembly, yet their influence on the possible cross-assigned reads and accuracy of serotype prediction are unknown. With DNA extraction method generating regular read length of genome DNA (mean read length around 8 Kbp), we recommend using assembled genomes for serotype prediction through SeqSero2 with multiplex ONT sequencing data, if more rapid serotype prediction enabled by raw reads input [∼5 s/million bases using SeqSero2 nanopore workflow (Zhang et al., 2019)] is not the primary concern.

### Influence of Sequencing Quality and Barcode Quality on the Accuracy of Serotype Prediction

Accurate serotype prediction of the two isolates noted above (FSL R9-3346, FC10-BC01, serotype Typhimurium, and isolate FSL S5-0658, FC19-BC03, serotype Senftenberg) was obtained when sequencing quality score was raised to ≥13 and qcat barcode score was set at ≥60 (default setting) using ONT raw reads as input of SeqSero2. However, this approach removed about 60% of the raw sequence data. Setting the quality score to ≥13 may have removed most of the error-causing reads from the data set, as the average sequencing quality score of the possible cross-assigned reads was 12.93. When the sequencing quality score was set to ≥14, more than 90% of the raw sequence data were lost. Consequently, the depth of genome coverage of some of the other isolates multiplexed in the same flow cell dropped to below 10×, which led to serotype prediction errors for these isolates due to low genome coverage.

When sequencing quality score was set at 7 (default setting) for filtering raw reads, and qcat barcode score was raised to ≥90 at the same time, accurate serotype prediction was obtained for these two samples by using raw reads with SeqSero2. However, these settings still resulted in loss of more than 90% of the raw sequence data, and reduction of the depth of genome coverage of some of the other isolates in the same flow cell to below 10×, again leading to serotype prediction errors for these isolates.

In summary, raising the quality score of raw reads to ≥13 improved serotype prediction accuracy for the isolates tested in the current study, while removing more than half of the total sequencing data. On the other hand, raising the barcode score of raw sequencing reads did improve prediction accuracy, but resulted in exclusion of 90% of the raw sequencing data, thus introducing errors due to lack of sequencing depth for some of the isolates multiplexed in the same flow cell. This approach could be used to avoid errors caused by cross-assigned reads when sequencing data depth permits, or where sequencing time length is not the primary concern. Xu et al. (2018) found that chimeric reads and low-barcode-score reads were the main causes of cross sample contamination in their data set. In contrast, we found the error-causing reads were of high barcode quality and without evidence of having internal adaptors. Removing all the reads with middle adaptors from the raw sequencing data did not alter the prediction results generated by using ONT raw reads (data not shown). This discrepancy in the cause of cross contamination may be attributed to the difference in library preparation and barcoding kits used in the current study and Xu’s study (Xu et al., 2018).

### Recommendation for Cost-Effective Multiplexing Strategy and Limitations of the Current Study

Multiplexing has the advantage of higher time and cost efficiencies compared to sequencing single isolates, particularly in a practical scenario where large numbers of samples are routinely analyzed. The sequencing kit (RBK004) used in this study has the capability of multiplexing up to 12 different isolates in one flow cell, which will yield considerable costs savings relative to sequencing a single isolate on a flow cell. Multiplexing three isolates could reduce consumable cost per isolate by 64% compared to the cost of sequencing a single isolate, and the cost reduction can be increased to 87% when ten isolates are multiplexed. However, the total sequencing time needs to be increased above the 1∼2 h required for single isolate sequencing, s so that sufficient data are obtained for each multiplexed isolate to allow for correct serotype prediction. Also, the unevenness of data yield between each multiplexed isolate increases as the number of isolates being multiplexed increases. The total number of multiplexed isolates, therefore, should be carefully considered. In this study 50 × depth of genome coverage (∼ 5 Gbp of raw data) could be obtained within an average of 6.0 h of ONT sequencing when multiplexing five isolates, while multiplexing seven isolates took an average of 19.5 h to reach equivalent depth (Table 4). Hence multiplexing seven to ten isolates resulted in only a small cost benefit compared with multiplexing five isolates (Table 4). Moreover, our previous study showed that both the data yield and quality started to decline after 12 h of sequencing on the flow cells of GridION, and the flow cells usually showed remarkably low numbers of active pores after 48 h of sequencing (Xu et al., 2020). These data suggest that multiplexing five isolates is likely to be more efficient overall, than multiplexing greater numbers of isolates. Total turnaround time is of critical importance in the practical application of serotype prediction in the food industry; multiplexing five isolates will allow serotyping results to be obtained within one day. Increasing the number of isolates being multiplexed resulted in increased sequencing time, and more total time for DNA extraction, library construction and data analysis (Table 4).

**TABLE 4:** Summary of turnaround time and cost for each multiplexing strategy.

**Multiplexing isolate number**	**1**	**3**	**4**	**5**	**7**	**10**
Cost per isolate^a^	$910	$330	$255	$210	$159	$120
Average sequencing time to obtain at least 50 × depth of genome coverage for each multiplexed *Salmonella* isolate	<1.0 h	2.9 h	4.0 h	6.0 h	19.5 h	>25.1
Data analysis time	<1.0 h	<2.0 h	<2.0 h	<2.0 h	<3.0 h	<3.0 h
DNA extraction + quality control	3.5 h	3.5 h	3.5 h	4.0 h	4.5 h	4.5 h
Library construction	1.0 h	1.5 h	1.5 h	1.5 h	2.0 h	2.0 h
Total time	6.5 h	9.9 h	11.0 h	13.5 h	29.0 h	34.6 h

*^a^Cost per isolate was calculated based on the market price of DNA extraction and ONT consumables in China in Dec. 2019.*

It is not known if certain strains of *Salmonella* would alter the accuracy of serotyping under the recommended settings, as only Ten serotypes were involved in the current study. Further validation with more *Salmonella* serotypes is necessary to operationalize the serotyping of *Salmonella* through multiplex ONT WGS, for example in the food industry or in public health.

## Conclusion

This study demonstrated that accurate serotype prediction results could be obtained when multiplexing five or less *Salmonella* isolates with an average of 6 h of multiplex ONT sequencing, where each multiplexed isolate received at least 50 × depth of genome coverage of sequencing data after demultiplexing. Multiplexing up to five isolates in one flow cell is recommended to achieve high coverage of the genome and high accuracy of prediction within one day. Multiplexing five isolates results in a cost reduction to 23% of the cost of ONT sequencing of a single isolate per flow cell. Our study helps to identify the optimal combinations of isolate multiplexing number and sequencing time to achieve the most accurate, rapid, and cost-efficient *Salmonella* serotype prediction with multiplex ONT sequencing. This study also sets a starting point for the future validation of multiplex ONT WGS as a cost-efficient, rapid *Salmonella* confirmation, and serotype classification tool for the food industry.

## Data Availability Statement

The datasets presented in this study can be found in online repositories. The names of the repository/repositories and accession number(s) can be found below: https://www.ncbi.nlm.nih.gov/, PRJNA694442.

## Author Contributions

ST and CG: conception and design of the work. XW, ST, and HL: data collection, data analysis, and drafting the article. CG, FX, GZ, SL, XD, MW, AS, and RB: critical revision of the article. All authors contributed to the article and approved the submitted version.

## Conflict of Interest

The authors declare that the research was conducted in the absence of any commercial or financial relationships that could be construed as a potential conflict of interest.

## Footnotes

1 https://www.illumina.com/systems/sequencing-platforms.html

2 https://nanoporetech.com/products

3 https://github.com/denglab/SeqSero2

4 https://github.com/nanoporetech/qcat

5 https://github.com/rrwick/Porechop

6 https://github.com/rrwick/Filtlong

7 https://github.com/denglab/SeqSero2/releases

8 https://github.com/ruanjue/wtdbg2/releases/tag/v2.4

9 https://github.com/isovic/racon/releases

10 https://blast.ncbi.nlm.nih.gov/Blast.cgi

11 https://nanoporetech.com/sites/default/files/s3/literature/microbial-genome-assembly-workflow.pdf

12 https://www.illumina.com/science/education/minimizing-index-hopping.html
