# Supplementary material for: Evaluation of Salmonella Serotype Prediction With Multiplex Nanopore Sequencing
Source: Front Microbiol. 2021 Mar 10;12:637771. doi: 10.3389/fmicb.2021.637771 (PMC7987803; doi:10.3389/fmicb.2021.637771)
Supplement: Supplementary file 1 [file Table_1.pdf]

**Supplementary Table 1**. The proportion of sequencing data yield of each barcode within each flow cell

| Pooling strategy | Flow cell ID | BC01 | BC02 | BC03 | BC04 | BC05 | BC07 | BC08 | BC09 | BC10 | BC11 | Mis-assigned reads | Non-assigned reads |
|---|---|---|---|---|---|---|---|---|---|---|---|---|---|
| 3 isolates | FC01 | 29.36% | 34.95% | 27.85% | - | - | - | - | - | - | - | 0.03% | 7.82% |
| | FC02 | 21.89% | 38.82% | 31.34% | - | - | - | - | - | - | - | 0.03% | 7.92% |
| | FC03 | 23.74% | 32.10% | 33.92% | - | - | - | - | - | - | - | 0.04% | 10.20% |
| | FC04 | 38.65% | 27.02% | 25.93% | - | - | - | - | - | - | - | 0.03% | 8.37% |
| | FC05 | 37.69% | 32.90% | 20.61% | - | - | - | - | - | - | - | 0.03% | 8.77% |
| 4 isolates | FC06 | 24.86% | 27.02% | 10.85% | 29.31% | - | - | - | - | - | - | 0.03% | 7.93% |
| | FC07 | 27.00% | 22.48% | 19.38% | 23.29% | - | - | - | - | - | - | 0.03% | 7.82% |
| | FC08 | 25.38% | 21.89% | 19.98% | 24.77% | - | - | - | - | - | - | 0.03% | 7.95% |
| | FC09 | 21.06% | 27.26% | 23.02% | 20.84% | - | - | - | - | - | - | 0.03% | 7.79% |
| | FC10 | 19.94% | 21.04% | 23.76% | 27.95% | - | - | - | - | - | - | 0.03% | 7.27% |
| 5 isolates | FC11 | 15.07% | 21.60% | 13.08% | 15.42% | 23.75% | - | - | - | - | - | 0.04% | 11.03% |
| | FC12 | 15.94% | 21.16% | 12.98% | 17.41% | 23.64% | - | - | - | - | - | 0.04% | 8.84% |
| | FC13 | 17.22% | 19.87% | 13.56% | 16.98% | 22.51% | - | - | - | - | - | 0.04% | 9.82% |
| | FC14 | 18.67% | 24.83% | 17.54% | 22.37% | 9.03% | - | - | - | - | - | 0.03% | 7.54% |
| | FC15 | 15.36% | 23.67% | 13.70% | 16.20% | 22.15% | - | - | - | - | - | 0.03% | 8.88% |
| 7 isolates | FC16 | 14.77% | 15.76% | 13.40% | 17.04% | 14.86% | 4.32% | 12.80% | - | - | - | 0.03% | 7.02% |
| | FC17 | 14.21% | 15.52% | 12.68% | 18.98% | 16.45% | 3.81% | 11.04% | - | - | - | 0.02% | 7.29% |
| | FC18 | 14.45% | 18.30% | 8.89% | 20.24% | 15.92% | 5.70% | 9.25% | - | - | - | 0.02% | 7.23% |
| | FC19 | 14.58% | 18.47% | 6.45% | 21.63% | 13.87% | 3.16% | 14.16% | - | - | - | 0.03% | 7.65% |
| | FC20 | 27.24% | 14.09% | 9.40% | 15.50% | 12.47% | 4.25% | 10.19% | - | - | - | 0.02% | 6.84% |
| 10 isolates | FC21 | 6.41% | 11.56% | 9.64% | 14.78% | 7.00% | 8.98% | 10.44% | 6.90% | 5.03% | 11.29% | 0.01% | 7.95% |
| | FC22 | 5.95% | 13.09% | 7.24% | 16.72% | 2.11% | 10.97% | 8.26% | 8.71% | 5.75% | 13.48% | 0.01% | 7.69% |
| | FC23 | 9.97% | 11.23% | 11.24% | 14.07% | 6.49% | 8.81% | 10.42% | 6.52% | 5.45% | 8.12% | 0.02% | 7.66% |
| | FC24 | 10.16% | 11.46% | 8.45% | 17.46% | 10.19% | 10.66% | 4.36% | 4.33% | 3.88% | 12.21% | 0.01% | 6.83% |
| | FC25 | 9.66% | 12.63% | 11.52% | 15.22% | 8.74% | 7.51% | 7.48% | 6.58% | 3.81% | 10.37% | 0.01% | 6.47% |
