# Supplementary material for: Evaluation of Salmonella Serotype Prediction With Multiplex Nanopore Sequencing
Source: Front Microbiol. 2021 Mar 10;12:637771. doi: 10.3389/fmicb.2021.637771 (PMC7987803; doi:10.3389/fmicb.2021.637771)
Supplement: Supplementary file 2 [file Table_2.pdf]

Supplementary Table 2. Identification of cross-assigned reads

Flow cell & Barcode ID	Isolate ID	Recorded serotype (antigen profile)	SeqSero2 predicted serotype using raw reads (antigen profile)	Subject seq-id	Read length	Read Quality	Barcode (Score)	Query seq-id	Alignment length	Identity (percentage)	Number of identical matches	Query coverage per subject	Query coverage per HSP
FC10-BC01	FSL R9-3346	Typhimurium (1,4,[5],12:i:1,2)	Stratford (1,3,19,:i:1,2)	Read No. 1	11132	12.6	BC01 (95.8)	O-9_46_wzy__1191	1206	94.28	1137	100	100
								O-3_10_wzx__1539	1553	94.33	1465	100	100
FC19-BC03	FSL S5-0658	Senftenberg (1,3,19:g,[s],t:-)	This predicted serotype is not in the Kauffman-White scheme (1,3,19:g,s,t:e,n,z15)	Read No. 2	6279	13.6	BC03 (100)	fljB_e_n_z15_Braenderupstr_AOZM01000055_e__1496	1505	94.22	1418	100	100
								fljB_e_n_z15_from-Chailey_SRR1049702_e__1506	1515	94.13	1426	100	100
								fljB_e_n_z15_Mbandakastr_AMRS01000032_e__1496	1505	95.28	1434	100	100
								fljB_e_n_z15_Salmonella.enterica_Uno_AY353325_e__1506	1515	94.26	1428	100	100
				Read No. 3	15476	12.6	BC03 (100)	fljB_e_n_z15_Braenderupstr_AOZM01000055_e__1496	1518	93.15	1414	100	100
								fljB_e_n_z15_Mbandakastr_AMRS01000032_e__1496	1518	94.2	1430	100	100
